# Spontaneous Intracranial Hypotension: Case Study and Review of the Literature

**DOI:** 10.7759/cureus.7018

**Published:** 2020-02-17

**Authors:** Stacey Podkovik, Samir Kashyap, Sruthi Bonda, Ira Bowen, Mark Calayag

**Affiliations:** 1 Neurosurgery, Riverside University Health System Medical Center, Moreno Valley, USA; 2 Neurosurgery, Touro University College of Osteopathic Medicine California, Vallejo, USA; 3 Neurosurgery, Kaiser Permanente Fontana Medical Center, Fontana, USA

**Keywords:** spontaneous intracranial hypotension, cerebrospinal fluid leak, spontaneous subdural hematoma, cerebellar mutism, orthostatic headaches, brain sagging, pachymeningeal enhancement

## Abstract

Spontaneous intracranial hypotension (SIH) is a pathology characterized by orthostatic headaches, diffuse pachymeningeal enhancement on magnetic resonance imaging (MRI), and low to normal cerebrospinal fluid (CSF) pressures.

We present the case of a 46-year-old male with refractory postural headaches, found to have a diffuse CSF leak throughout the cervicothoracic (C1-T12) spine. His neurological status declined rapidly to a Glasgow Coma Scale (GCS) of eight, necessitating bilateral subdural drain placement. Despite an overall brisk neurologic recovery, the patient remained unable to speak for nearly a week after the return of the remainder of his function. This raised the concern for possible cerebellar mutism.

We review the multiple modalities used in this patient’s treatment and explore possible explanations for the failure of initial therapy. The placement of bilateral subdural drains was a temporizing measure to treat the patient’s neurologic decline, but it was likely the epidural blood patch with prolonged bedrest that hastened the patient’s recovery. His speech function also returned with time and repeated therapy.

## Introduction

Spontaneous intracranial hypotension (SIH) is traditionally defined by a cerebrospinal fluid (CSF) pressure of 6 cmH20 or less when a patient is in the lateral decubitus position [1]. This phenomenon is not as rare as once believed, occurring once for every two reported spontaneous subarachnoid hemorrhages or approximately five per 100,000 cases. It is twice as common in females and is usually diagnosed in the fourth or fifth decade of life, peaking at age 40 [2-3]. SIH results from a CSF leak secondary to structural weakness in the dura, either at the cervicothoracic junction or the thoracic spine. The size of the leak can vary from a small leak during a Valsalva maneuver, to a large amount leaking spontaneously [3]. It is typically secondary to dural weakness compounded by exertional activity, which can be associated with connective tissue disorders such as Marfan syndrome [3]. Dural injury can also occur from a congenital osseous spur or acquired degenerative disc disease that punctures the dura [3]. The most commonly seen symptom of SIH and a CSF leak is a postural headache that exacerbates within 15 minutes of standing and improves within 30 minutes of lying down [3]. Cranial nerve abnormalities, such as diplopia, hearing difficulties, trigeminal neuralgia, and facial weakness, have also been reported in SIH [2,4-6]. Although patients may develop subdural hematomas or hygromas, management is centered around the treatment of the underlying cause rather than the hematoma or hygroma. We present a case of a patient with refractory SIH resulting in severe neurological compromise and its subsequent management.

## Case presentation

Presentation

A 46-year-old Vietnamese male presented to his primary care physician after a severe coughing fit following an upper respiratory infection. His initial complaints were positional headaches and subsequent neck pain. An extensive headache workup consisting of tension headaches, influenza screens, and migraine evaluations was negative, and his pain was refractory to various medications such as fluticasone, cetirizine, sumatriptan, nortriptyline, methocarbamol, and ibuprofen.

**Figure 1:**
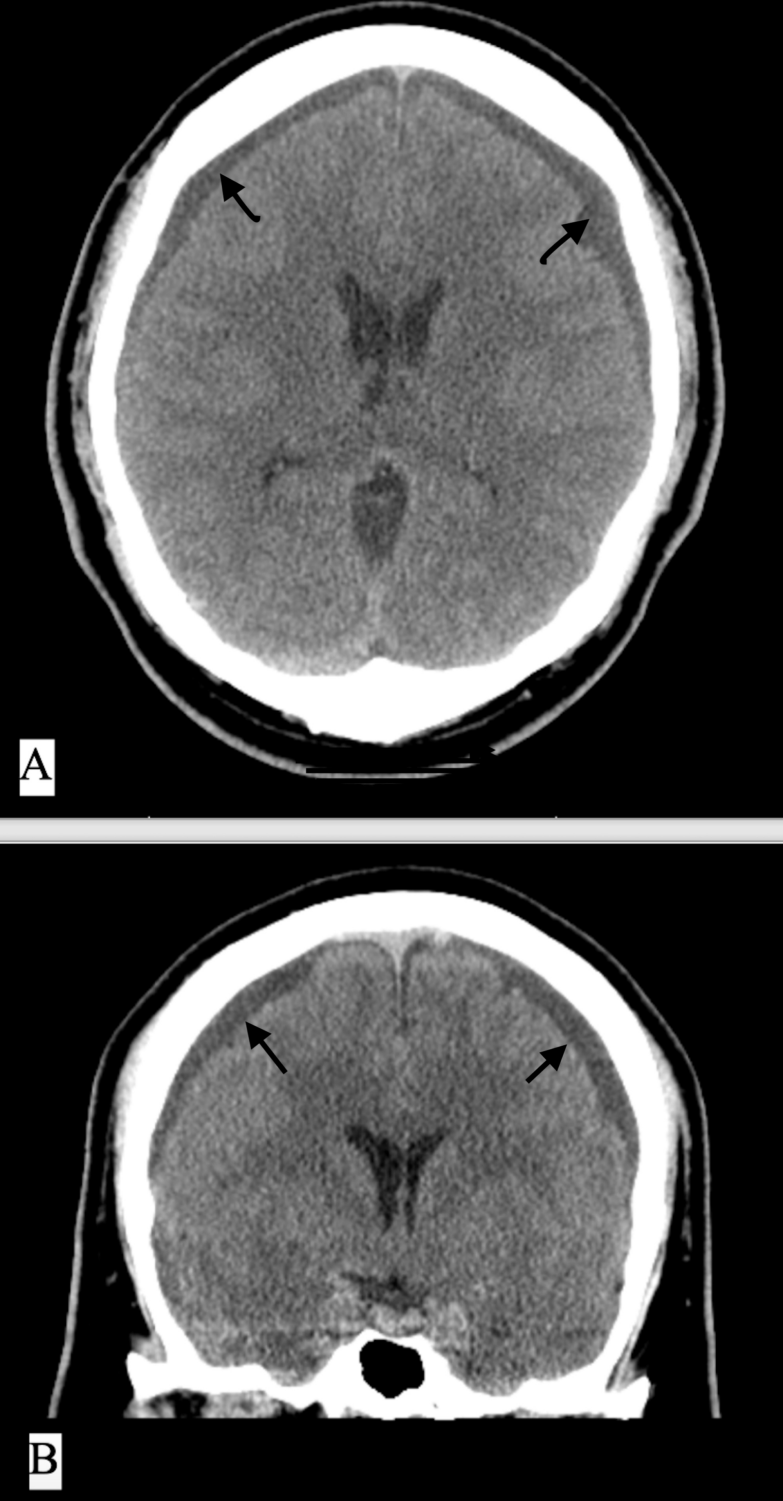
Initial CT imaging during headache workup; evidence of chronic bilateral subdural fluid collections A) CT of the head, axial cut, non-contrast; B) CT of the head, coronal cut, non-contrast

**Figure 2:**
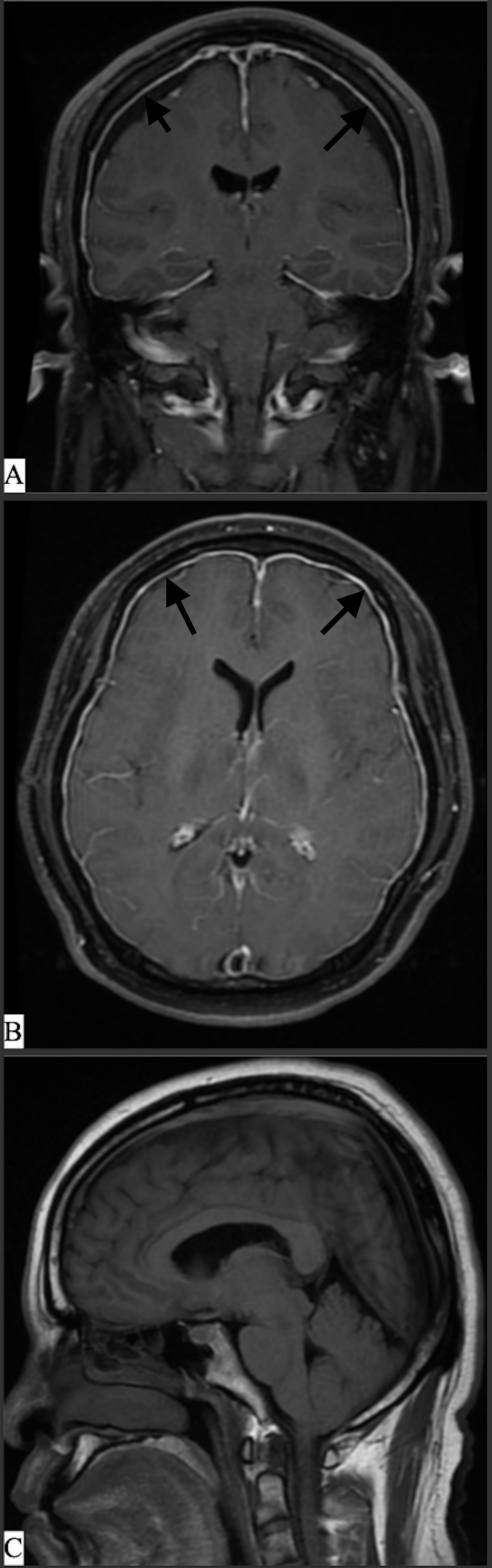
Initial MRI brain A) Initial MRI brain with gadolinium, coronal view. The patient demonstrated chronic bilateral subdural hematomas. B) MRI brain with gadolinium, axial view, demonstrating diffuse, non-nodular, pachymeningeal enhancement. C) MRI brain with gadolinium, sagittal. MRI imaging during initial workup.

**Figure 3:**
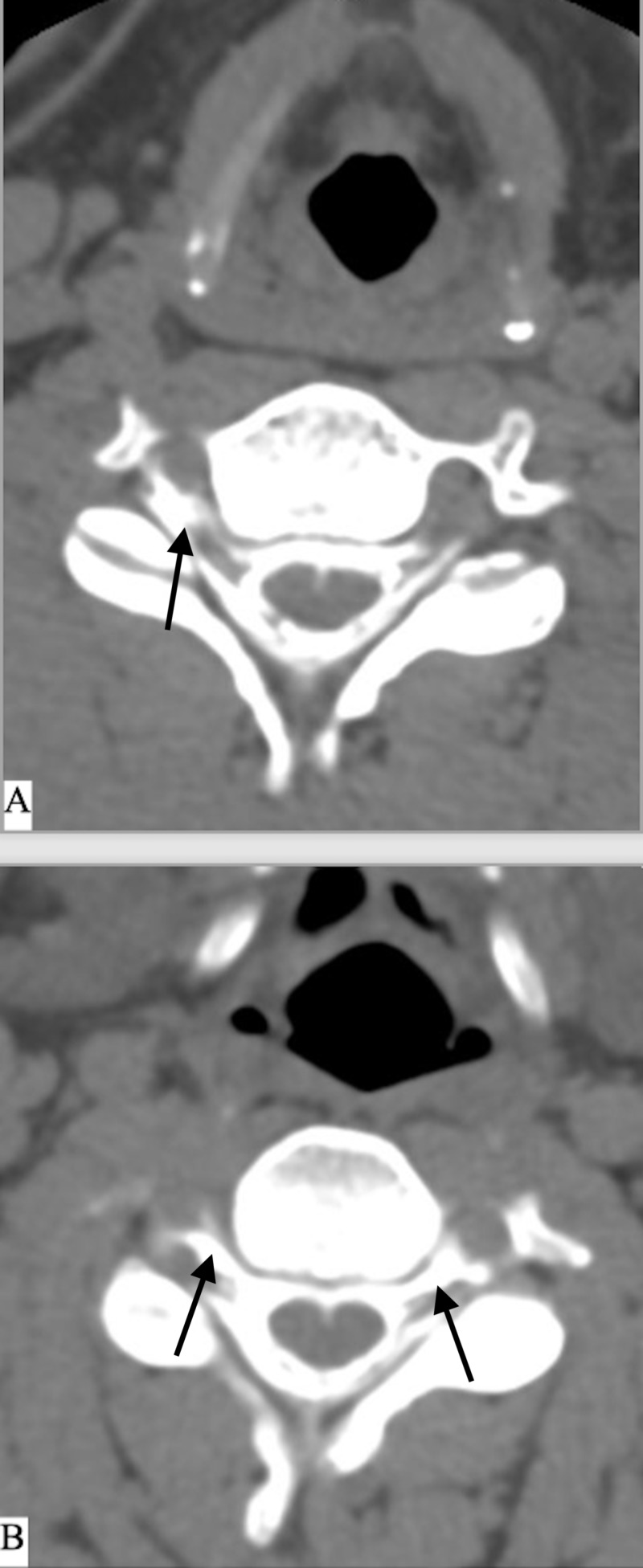
CT myelogram of the cervical spine A) Depicts the C4 level with contrast extravasation along the bilateral nerve roots. B) This is the C5-6 level with contrast extravasation predominantly along the right nerve root.

Two months after symptom onset, a non-contrast computed tomography (CT) of the head demonstrated bilateral chronic subdural hematomas (cSDH), 7 mm on the right and 8 mm on the left (Figure 1). MRI brain with contrast demonstrated diffuse pachymeningeal enhancement without nodularity, tonsillar herniation, and a decreased pontomedullary distance of 4 mm (Figure 2). Subsequent CT myelogram (Figure 3) showed diffuse contrast extravasation surrounding the bilateral nerve roots from C1-T12 but most prominently from C4-7 on the right side. The patient underwent two blood patches three days apart, neither of which provided meaningful relief.

Clinical course

**Figure 4:**
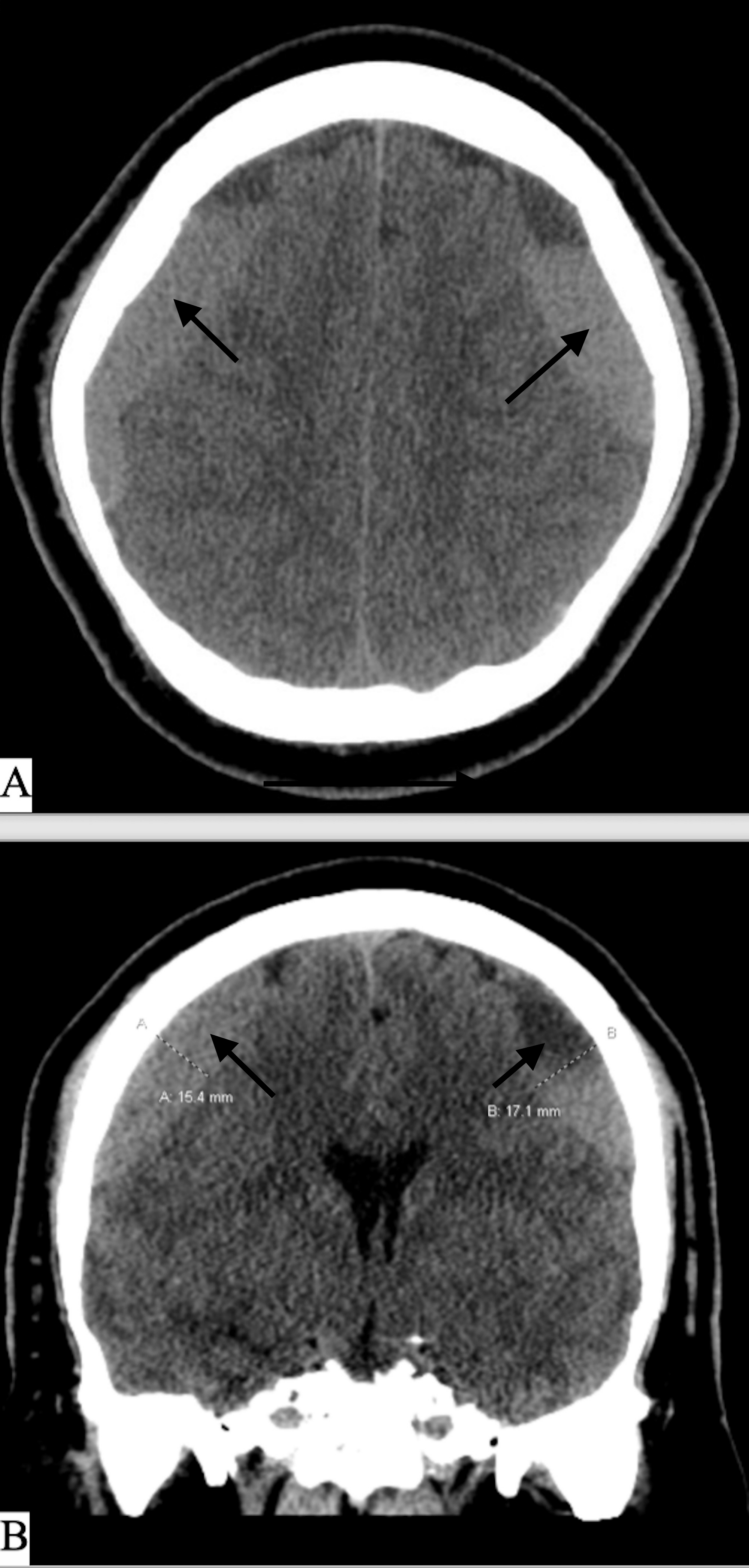
CT head on day of admission A) Axial CT head non-contrast on day of admission. B) Coronal CT head non-contrast on day of admission, showing enlargement of the bilateral subdural hematomas with mixed density components.

**Figure 5:**
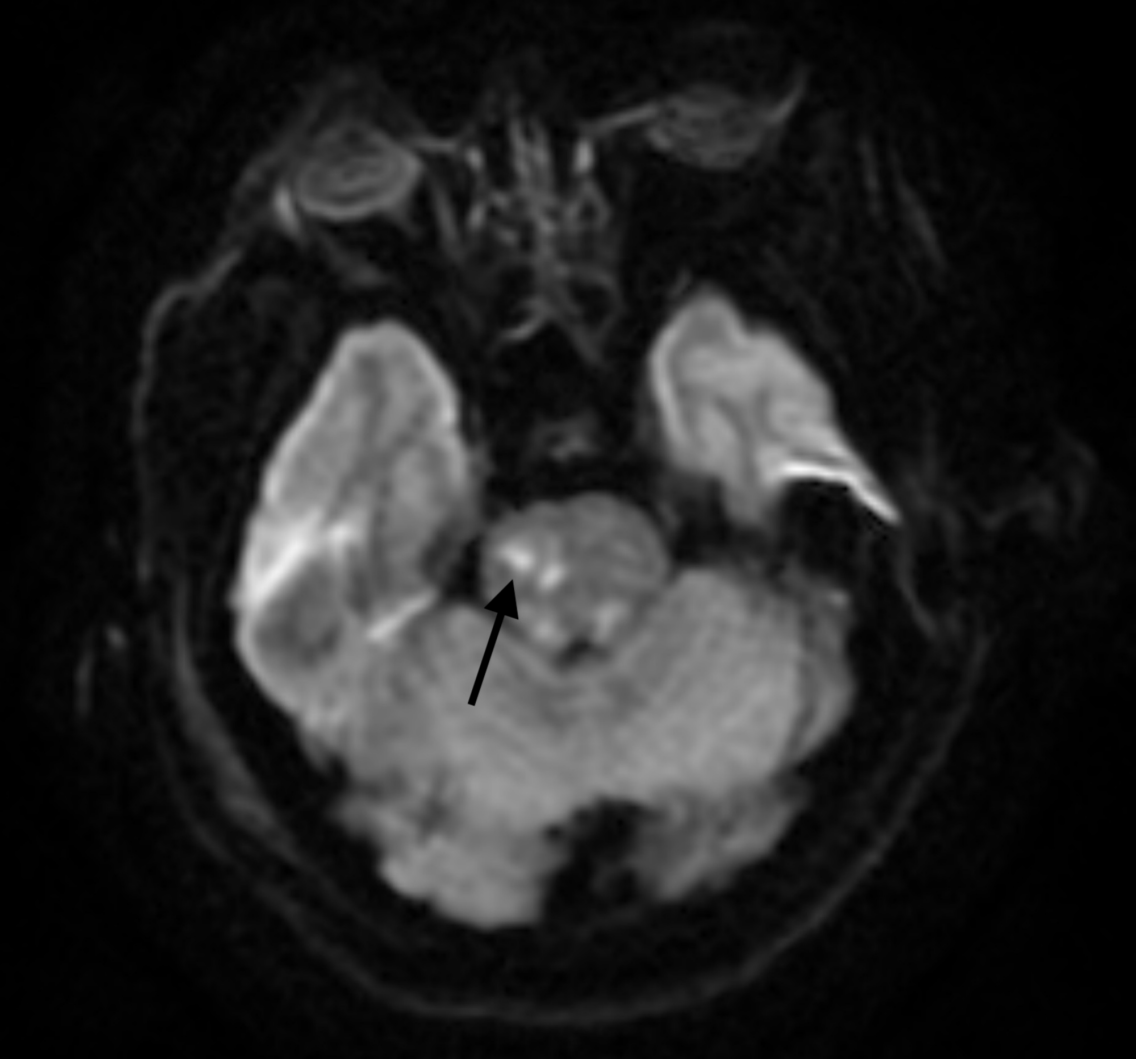
MRI brain after drain removal MRI of the brain without gadolinium. Imaging completed after removal of both subdural drains. There is evidence of two, new, right-sided, punctate, pontine ischemic strokes.

**Figure 6:**
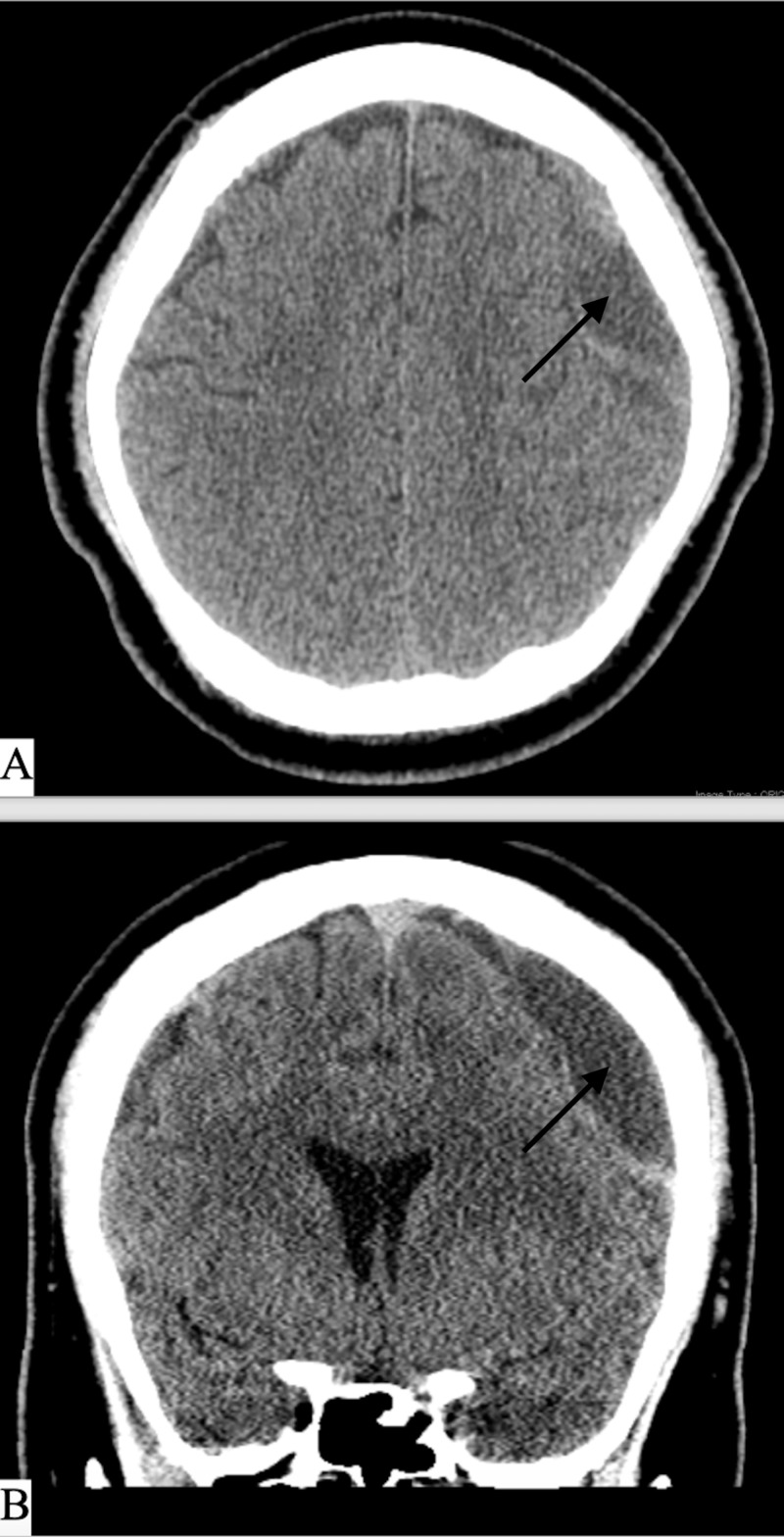
CT head at one-month postop visit A) CT of the head without contrast, axial cut. Obtained at the patient’s one-month follow-up visit. Recurrence of the left-sided subdural fluid collection. B) CT of the head without contrast, coronal cut. Evidence of left hypodense subdural fluid collection; likely hematoma.

The patient’s wife reported that he was becoming increasingly confused and lethargic a week after the second blood patch. On initial neurosurgical evaluation, he had a Glasgow Coma Scale (GCS) score of 14, was alert and oriented, but mildly confused regarding his current overall health status. Repeat CT of the head without contrast and MRI of the brain with gadolinium demonstrated increased bilateral cSDH to approximately 1.5 cm bilaterally (Figure 4). On hospital day two, the patient declined to a GCS 8 (motor 5, verbal 2, eyes 1). The patient’s unique speech deficit was not apparent at first due to his overall poor neurologic status. Given his rapid decline, a right-sided subdural drain was placed, followed by a left-sided drain two days later. The patient’s neurologic alertness slightly improved after drain placement, but he remained non-verbal. Repeat MRI of the brain after drain removal re-demonstrated the same diffuse pachymeningeal enhancement, however, new bilateral punctate pontine acute ischemic infarcts were noted (Figure 5). After drain removal, the patient underwent a repeat blood patch at T12-L1 and was kept flat for four days. The patient experienced a brisk neurologic recovery over the next five days. He readily followed commands, including oral movements; however, the patient appeared to have a speech apraxia. With repeated therapy and time, the patient’s speech returned over a two-week period. The patient was subsequently discharged home. At his one-month follow-up, the patient remains neurologically at his baseline. Repeat CT head again shows bilateral subdural hygromas, worse on the left as compared to his discharge imaging (Figure 6).

## Discussion

Spontaneous CSF leaks are most commonly found in the thoracic spine followed by the cervicothoracic junction [3,7]. Our patient demonstrated a diffuse CSF leak at almost every level of his cervical and thoracic spines, most severe at the right C4-7 nerve roots (Figure 3). Once a diagnosis of spontaneous cerebrospinal fluid (CSF) leak had been established, further questioning elicited that he did not have any traumatic events except for an extremely strong cough. This increased his intra-thoracic, intra-abdominal, and intracranial pressures. Due to his rapidly declining mental status, it became necessary to place bilateral subdural drains, as it appeared that the bilateral subdural hematomas were the cause of his decline. The drains were kept to gravity in order not to propagate a continued negative pressure. The drains were removed after five days following the patient’s gradual improvement in neurologic exam.

Currently, there is limited consensus on the treatment approach for SIH complicated by SDH that vary from craniotomies for hematoma evacuation to solely directing treatment at the underlying dural defect [8]. Undiagnosed or inadequately treated SIH results in persistent SDH with high recurrence rates. Growing evidence supports treatment focused on the correction of the CSF leak without hematoma evacuation; however, Ferrante et al. reported that three out of 18 patients required drainage due to a significant mass effect [1,8-11]. Of note, one of the greatest challenges in diagnosis sometimes resides in distinguishing the difference between whether the symptoms are due to hypotensive versus compressive forces.

Most patients tend to have sufficient recovery from conservative measures such as rest and hydration. When these measures fail, a trial of an epidural blood patch (EBP) is attempted. Our patient likely did not recover from his outpatient EBP for multiple reasons. His CSF leak was localized primarily at the cervicothoracic junction, whereas the EBPs were done at L4-5 on the first attempt and T12-L1 on the second attempt. The patient lay flat for two hours after the procedure prior to going back to his normal daily routine. Although routinely not performed, it has been reported that an EBP is more effective when done closer to the site of the CSF leak [12]. In trained hands and with fluoroscopic or ultrasonic guidance, it is possible to do an EBP closer to the site of the leak. It is also likely that lying flat for two hours was not enough for the blood patch to sufficiently cover the appropriate levels. After the third blood patch, performed at T12-L1, the patient was kept flat for nearly 72 hours before beginning to mobilize him. Surgical intervention in this patient would not have been practical, as localizing a site to repair was not possible and commonly is not required.

As seen in our case, orthostatic headaches and neck pain are the most common presenting symptoms in spontaneous intracranial hypotension (SIH) [1,7]. Headaches are usually localized to the occipital-nuchal or frontal areas and are thought to be caused by the inferior displacement of the brain from a decreased volume, which puts tension on pain-sensitive structures [1,3]. Women are affected twice as often as males, with the symptoms typically presenting in the fourth or fifth decade of life [2-3]. Ferrante et al. looked at 18 patients over the course of nine years and found that orthostatic headaches were the initial presenting symptom in almost 94% of their patients. This was associated with nausea and vomiting in four people, vertigo in eight people, and diplopia in six people [1]. Other presenting symptoms may include photophobia, phonophobia, tinnitus, altered balance, and local pain at the site of the leak [3].

It is believed that the most common cause of symptoms is a downward pulling force on intracranial structures and pain receptors [7]. Gravitational forces on the pituitary stalk have also been known to cause hyperprolactinemia and galactorrhea, as well as a decreased level of consciousness from the herniation of the diencephalon [2-3,13]. There are rare reports of Parkinsonism, ataxia, and cerebellar hemorrhage associated with SIH. Cases of frontotemporal dementia associated with spontaneous CSF leaks have also been reported.

In our patient’s presentation, his speech seemed to be the most perplexing, as it had a very protracted recovery when compared to his other symptoms. He was able to briskly follow commands shortly after the placement of the subdural drains and evacuation of the underlying hematomas. However, he remained with speech apraxia. There have been recent case reports, such as by Van Baarsen et al., which alluded to pontine infarcts that affect the dentate-rubro-thalamic tract within the superior cerebellar peduncle, leading to a case of cerebellar mutism [14]. The presence of bilateral pontine infarcts could have contributed, but it would have been unlikely for such a sudden improvement in his speech after several days. If the patient’s speech deficits were due to vascular ischemia, recovery would be a long process. We propose that brain sagging placed stress on his cerebellar pathways, leading to a form of cerebellar mutism. Although the mechanism is unknown, it is believed that cerebellar mutism is an extreme form of cerebellar dysarthria [15-16]. This sagging likely also decreased the amount of blood flow to the pons by placing a strain on the perforating arteries, leading to bilateral symmetric pontine infarcts.

**Figure 7:**
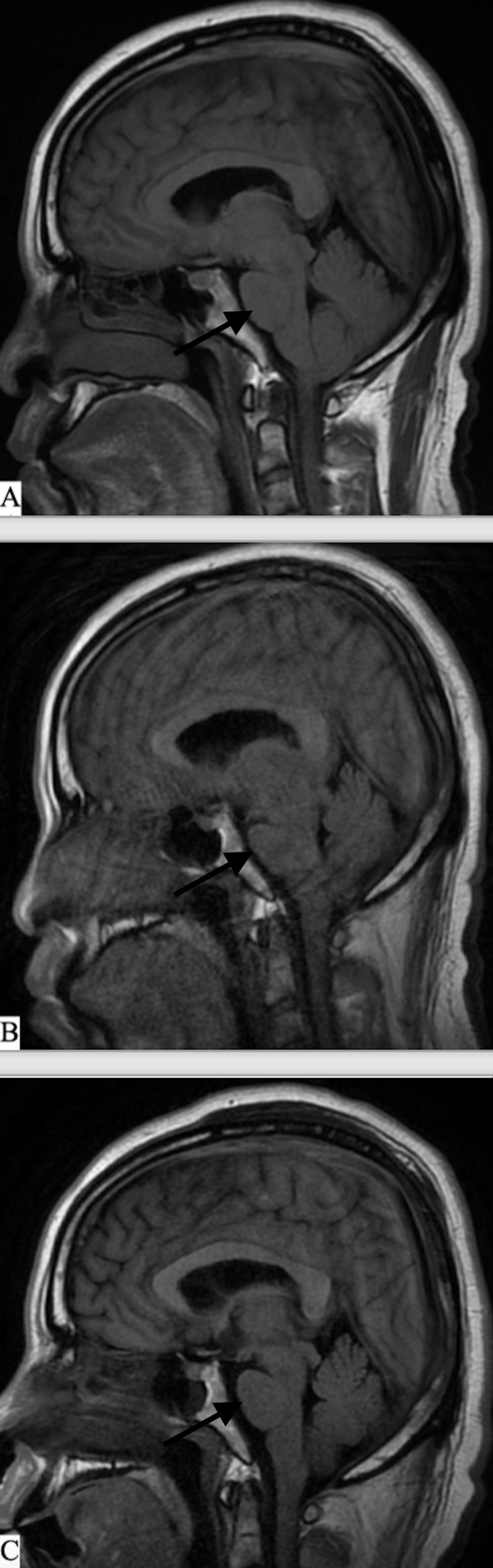
Pontomedullary junction on sequential MRI brain A) MRI brain with gadolinium (GAD), sagittal. MRI imaging during the initial workup. Re-demonstrated for comparison purposes. B) MRI brain without GAD, sagittal view. On admission with the start of altered mentation. Evidence of shortened pontomedullary distance. Prior to subdural drain placement. C) MRI brain without GAD, sagittal view. After the removal of subdural drains. Persistent bed rest and hydration. Improvement in the pontomedullary distance.

The pathognomonic findings on contrasted MRI brain are diffuse, smooth, pachymeningeal gadolinium enhancement (DPGE), and brain sagging. DPGE is typically due to the dilation of the dural veins secondary to negative pressure (Figure 2b) [1]. Ferrante et al. had 16 of 18 patients (88%) in their study that had evidence of DPGE [1]. Brain sagging is thought to be due to the downward displacement of the brain due to low CSF volume and subsequent buoyant forces. The relative amount of sagging relates to the severity of symptoms [17]. This brain sagging has been associated with multiple different presentations. Wicklund et al. looked at eight patients with radiographic evidence of brain sagging and noticed that all of the patients had symptoms related to the behavioral changes of disinhibition and apathy [18]. Concurrently with the behavioral changes, these same eight patients were noted to have increased daytime somnolence, one of the presenting symptoms of our reported patient. These symptoms, when severe enough, can mimic frontotemporal dementia [18]. Our patient was found to have all of the above imaging findings (Figure 7).

The presence of subdural hygromas or hematomas on imaging is estimated to be approximately 50% [19]. Subdural hygromas are generally recognized as thin, bilateral, and symmetrical, with minimal mass effect, if any, and are thought to be a compensatory response to CSF hypovolemia [20]. Subdural hygromas are self-limited, resolving within several weeks of successfully treating the underlying cause of SIH. Subdural hematomas (SDH) can also present bilaterally, but in contrast to hygromas, they tend to be asymmetric, are associated with significant mass effect, and account for 40%-45% of the subdural collections related to SIH [8]. SDH forms secondarily to the shearing force exerted on the bridging veins, caused by the characteristic brain sag and downward traction seen in SIH.

Schievink et al. described a group of SIH patients presenting with various minor trauma daily events, including roller coasters, childbirth, weight lifting, jogging, playing volleyball, sexual intercourse, and bumping into a table during a fight [2]. Another underlying cause of spontaneous CSF leaks includes connective tissue diseases such as Marfan Syndrome, Ehlers-Danlos syndrome, and autosomal dominant polycystic kidney disease. About 20% of patients have some Marfanoid features, and 40% have isolated joint hypermobility. The commonality in these patients is a defect in the microfibril gene [2,7]. There have also been some mentions of people without visible connective tissue disorders having family histories of retinal detachments, indicating some possible affected protein common to the retina and dura [3]. Less commonly, these spontaneous leaks can be caused by congenital anatomic bone spurs, leading to the perforation of the dura [2]. In this particular case, the patient did not have any pathognomonic features. One potential explanation is that leaks are commonly seen at nerve rootlets because they are branching points, which are commonly the weakest spots in structural integrity.

The patient’s recurrence of the left-sided subdural fluid collection at his one-month follow-up imaging is likely representative of a repeat CSF leak or evidence that the last blood patches were not entirely effective. The patient will need to be monitored closely with possible repeat EBPs to help close any leaks.

## Conclusions

Spontaneous cerebrospinal fluid leaks are commonly misdiagnosed. The brain sagging phenomenon associated with this entity leads to a myriad of neurological deficits and presenting symptoms. We discuss a unique example of this phenomenon leading to cerebellar mutism. It is important for clinicians to keep this diagnosis in mind when evaluating an abnormal presentation of unexpected neurologic findings, altered mentation, and especially with radiographic findings such as diffuse pachymeningeal enhancement.
